# The Healing Effect of Nettle Extract on Second Degree Burn Wounds

**Published:** 2015-01

**Authors:** Hosein Akbari, Mohammad Javad Fatemi, Maryam Iranpour, Ali Khodarahmi, Mehrdad Baghaee, Mir Sepehr Pedram, Sahar Saleh, Shirin Araghi

**Affiliations:** 1 Department of Plastic Surgery, Iran University of Medical Sciences, Tehran, Iran;; 2 Department of Plastic and Reconstructive Surgery, Burn Research Center, Hazrat Fatima Hospital, Iran University of Medical Sciences, Tehran, Iran; 3 Department of Pathology, Kerman University of Medical Sciences, Kerman, Iran;; 4 Department of Plastic Surgery, Kerman University of Medical Sciences, Kerman, Iran;; 5 Department of Surgery, Iran University of Medical Sciences, Tehran, Iran;; 6 Department of Surgery and Radiology, Faculty of Veterinary, Tehran University of Medical Sciences, Tehran, Iran

**Keywords:** Healing, Nettle, Burn, Wound, Silver sulfadiazine, Vaseline, Rat

## Abstract

**BACKGROUND:**

Numerous studies were carried out to develop more sophisticated dressings to expedite healing processes and diminish the bacterial burden in burn wounds. This study assessed the healing effect of nettle extract on second degree burns wound in rats in comparison with silver sulfadiazine and vaseline.

**METHODS:**

Forty rats were randomly assigned to four equal groups. A deep second-degree burn was created on the back of each rat using a standard burning procedure. The burns were dressed daily with nettle extract in group 1, silver sulfadiazine in group 2, vaseline in group 3 and without any medication in group 4 as control group. The response to treatment was assessed by digital photography during the treatment until day 42. Histological scoring was undertaken for scar tissue samples on days 10 and 42.

**RESULTS:**

A statistically significant difference was observed in group 1 compared with other groups regarding 4 scoring parameters after 10 days**.** A statistically significant difference was seen for fibrosis parameter after 42 days. In terms of difference of wound surface area, maximal healing was noticed at the same time in nettle group and minimal repair in the control group.

**CONCLUSION:**

Our findings showed maximal rate of healing in the nettle group. So it may be a suitable substitute for silver sulfadiazine and vaseline when available.

## INTRODUCTION

Burn is still regarded as one of the emergency medicine affecting both genders and all ages groups in both developed and developing countries leading to physical and psychological disabilities with an increasing trend in mortality and morbidity during pregnancy.^1^^,^^2^ Wound healing is widely discussed in the medical literature. In topical burn therapy, silver sulfadiazine was introduced as the gold standard having antibacterial properties too.^3^

Numerous studies were carried out to develop more sophisticated dressings to expedite healing process and diminish bacterial burden in wounds. Even medicinal plants were introduced in wound healing of burned injuries, traditional forms of medicine, especially herbal products, which have been employed for centuries in Africa and Asia are under scientific investigation for their roles in wound treatment.^4^^-^^7^ There are many reports confirming the use of medicinal plants for dressing of wounds who was described by Avicenna, the Persian physician (980-1037 AD) in his famous book, Canon of Medicine.^5^^,^^6^^,^^8^^-^^11^

Stinging nettle has been used for hundreds of years to treat painful muscles and joints, eczema, arthritis, gout, anemia and benign prostatic hyperplasia.^12^^-^^15^ Combudoron, composed of extracts from arnica and stinging nettle, was used for the treatment of partial thickness burns and insect bites in Europe. Combudoron seems to have positive effects on healing of grade 2 laser induced burns, which needs further investigation.^16^^,^^17^ This study assessed the effect of nettle extract on second-degree burns wound healing in rats in comparison with silver sulfadiazine and vaseline.

## MATERIALS AND METHODS

In a randomized clinical trial, 40 Wistar-albino male rats (average weight: 300-350 g, average age: 3-4 months) were randomly divided into 4 equal groups. Group 1 received topical nettle extract; group 2 was treated with topical silver sulfadiazine; for group 3, topical vaseline was applied and group 4 was considered as the control group with no medication. They were all maintained in a sheltered environment (temperature: 20-25˚ C and humidity: 65-75%) under the supervision of a veterinarian. During the experiment, the rats were fed with usual rat chow and tap water and each group was kept in a separate cage. Studies on all groups were done at the same time. All the rats were handled according to the ethical principles for animal experiments of International Council for Animal Protection. All the experimental procedures were approved by the research ethics committee of the university. 

The rats were anesthetized by xylazine (10 mg/kg) and ketamine hydrochloride (50-100 mg/kg). A standard 3^rd^ degree burn wound was produced using a hot plate with the same size about 20% total body surface area and at identical temperature as described before.^18^ Briefly, the skin on the dorsum was shaved with an electrical clipper. A deep second-degree burn wound was created with a hot plate (diameter: 4×2 cm) at an identical temperature (warmed 5 min and placed for 10 sec on the skin with an equal pressure). The burn area was treated with nettle extraction in group 1, silver sulfadiazine in group 2, vaseline in group 3 and without any intervention in group 4.

Response to treatment was assessed by digital photography twice per week under general anesthesia during the treatment until day 42. Histologic parameters (PMN infiltration, collagen deposition, fibrosis and angiogenesis) were assessed on biopsy specimens of the wound on days 10 and 42. Every specimen was taken under general anesthesia by a punch device which contained a part of wound and its surrounding skin. 

The specimens were stained with H&E and Masson’s trichrome. Histological criteria were defined as follows: Collagen: normal bundle: 2, disorganized/edematous: 1, amorphous: 0; PMN X40 field: 0-10: 2 , 11-40:1 ,>40: 0; angiogenesis to 3 degrees of mild, moderate and severe and fibrosis with thickness measurement of collagene bundle to 3 degrees of mild, moderate and severe.^19^

## RESULTS

One of the animals in silver sulfadiazine group and one animal in nettle group died on day 11 and one of the animals in vaseline group died on day 15. In the first biopsy (day 10) in nettle group compared with vaseline and control groups, a statistical significant healing effect was seen for the 4 scoring parameters (P<0.05)**. **In nettle group compared with silver sulfadiazine group, a significant difference was observed for 2 parameters of fibrosis and PMN infiltration**. **

**Fig. 1:**
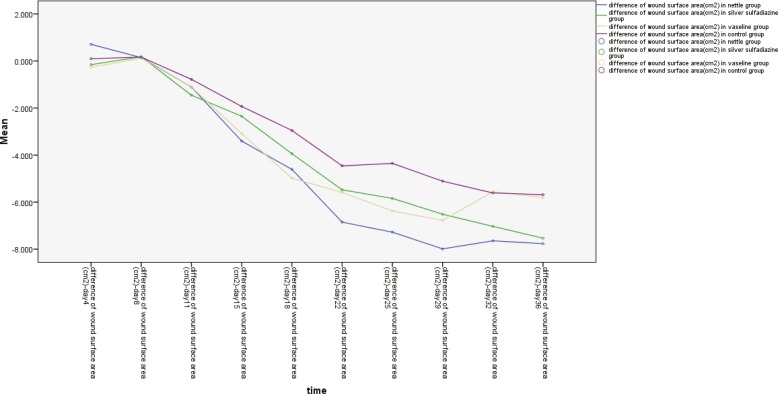
The wound surface area in terms of time in different groups

**Fig. 2:**
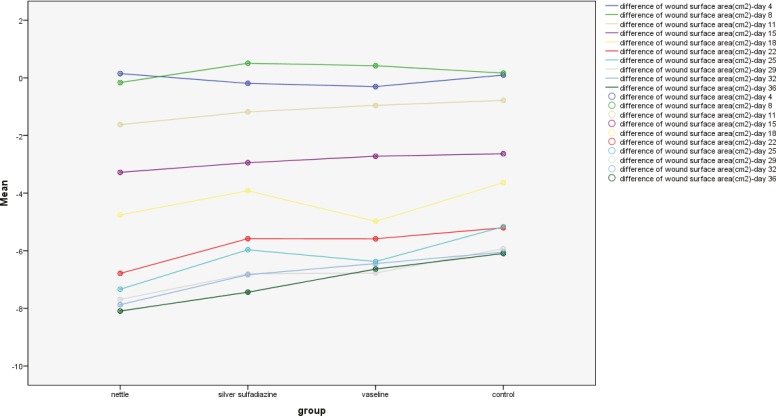
The wound surface area in terms of group at different time intervals.

**Fig. 3:**
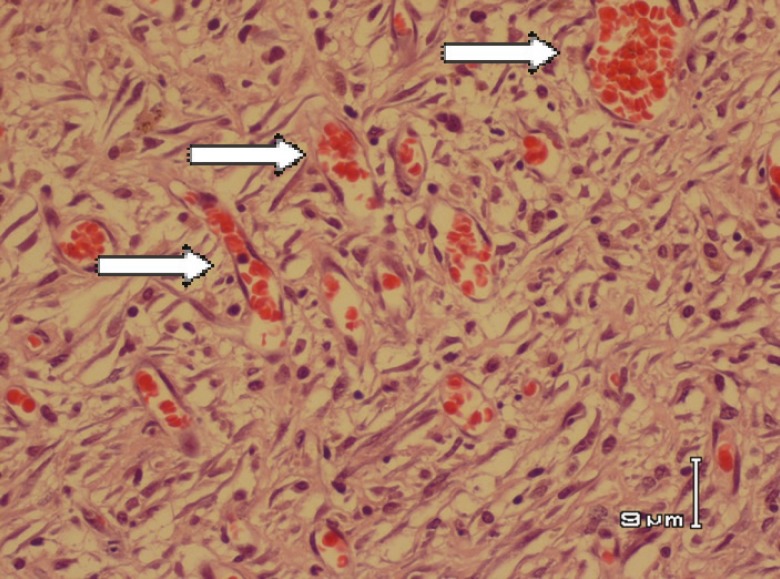
Severe angiogenesis (arrows) in dermis (H&E stain) in nettle group.

**Fig. 4:**
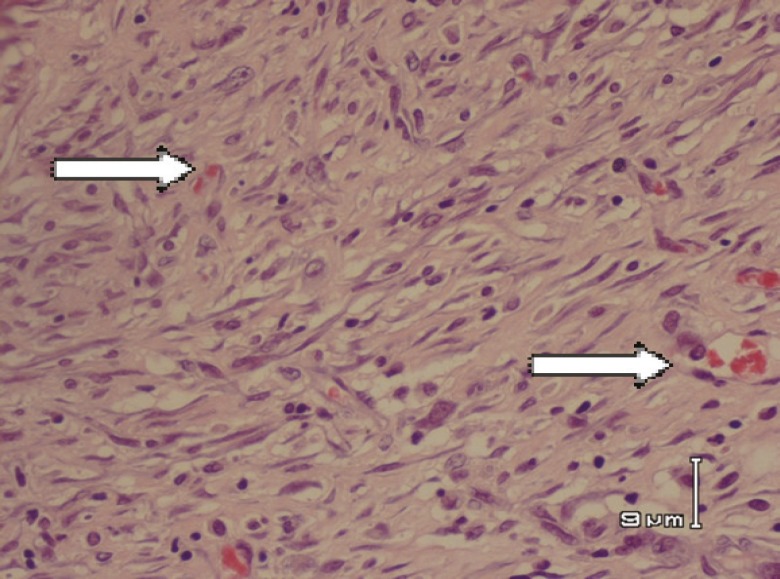
Mild angiogenesis (arrows) in dermis (H&E stain) in control group.

**Fig. 5:**
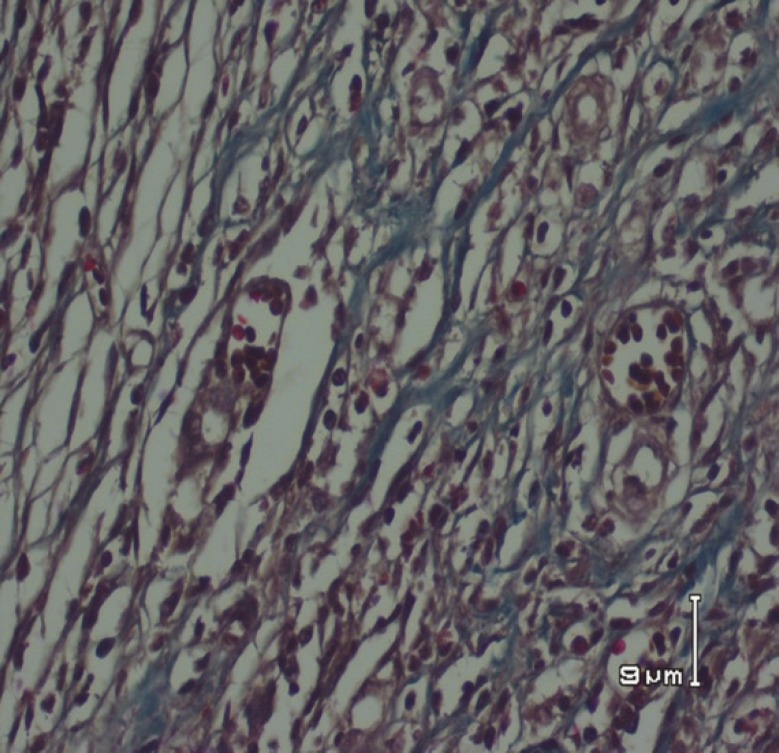
Dermis showing collagen deposition and dense fibrosis in nettle group (Masson’s trichrome stain).

In the second biopsy (day 42), for fibrosis parameter among 3 groups, namely, nettle/silver, silver/vaseline and silver/control, significant differences were found (Tables 1 and 2). For wound surface area at identical time intervals, maximal healing effect was seen in nettle group and minimal repair in the control group. Differences of wound surface area in the first 10 days were equal among all groups and a sharp slope of chart after day 10 showed more improvement in nettle group but was not statistically significant (Figures 1 and 2). A severe angiogenesis was present in dermis in Nettle group (Figure 3) while in control group, it was mild (Figure 4). Figure 5 denotes to dermis showing collagen deposition and a dense fibrosis in the nettle group. 

**Table 1:** Comparison of the healing effect of burn in all groups (P value<0.05: significant)

**Parameter**	**Fibrosis**		**Angiogenesis**	**PMN infiltration**	**Collagen deposition**
**Group**	**After 10 days**	**After 41 days**	**After 10 days**	**After 10 days**	**After 10 days**
Nettle+vaseline	N S	0.001	0.001	0.001	0.001
Nettle+control	N S	0.001	0.009	0.002	0.001
Nettle+SSD	0.001	0.01	NS	0.005	NS
Vaseline+SSD	0.009	0.008	0.007	NS	NS
Control+SSD	0.009	NS	NS	NS	NS
Vaseline+control	N S	NS	NS	NS	NS

P value<0.05: Significant; SSD=Silver sulfadiazine; NS=Not significant (P value>0.05).

**Table 2:** Comparison of the healing effect of burn between two time intervals

	**Collagen**		**PMN**		**Angiogenesis**		**Fibrosis**		**Epithelialization**	
**Time **	**Day 10**	**Day 41**	**Day 10**	**Day 41**	**Day 10**	**Day 41**	**Day 10**	**Day 41**	**Day 10**	**Day 41**
Value	20.906	4.270	17.811	0.299	15.605	4.333	21.113	12.950	4.880	1.012
Df	3	3	3	3	3	3	3	3	3	3
P value**	0.001	0.234	0.001	0.960	0.001	0.228	0.001	0.005	0.181	0.798

Df=Degree of freedom;

** P value<0.05: Significant

## DISCUSSION

Burns are among the most common and devastating forms of trauma. They are physical and chemical phenomena and cause many morbidities and mortalities in the world. The final goal of all of the current burn treatments is to accelerate skin healing and prevent wound infection.^8^^,^^14^^,^^20^ Cutaneous wound repair consists of orderly progression of events that establish the integrity of the damaged tissue. 

The sequence of events that repairs the damage is categorized into three overlapping phases: inflammation, proliferation, and tissue remodeling. The normal healing process can be impeded at any step along its path by a variety of factors that can contribute to impaired healing.^20^ The final step of the proliferative phase is epithelialization, which involves migration, proliferation, and differentiation of epithelial cells from the wound edges to resurface the defect. In open full thickness burn wounds, epithelialization is delayed until a bed of granulation tissue is established to allow for the migration of epithelial cells.^21^

Studies have demonstrated that burn infection is the main cause of mortality in patients with extensive burns. Therefore, many researchers have tried to achieve appropriate treatment methods to reduce the risk of wound infections and shorten the period of treatment in the patients with burn wounds. Some of these treatments involve using topical antimicrobial agents, which effectively reduce mortality rate of burns. 

One of these antimicrobial topical ointment is silver sulfadiazine with advantages such as easy and convenient use, not causing pain during administration, yielding low toxicity and sensitivity and having antibacterial effects, which have made it known as the gold standard among anti-microbial topical drugs for the patients with burns and turned it to the main consumed drug in the treatment of burn wounds around the world.^8^ Use of non-silver treatment led to shorter wound healing time, less dressing changes and shorter length of hospital stay, compared to silver sulfadiazine treatment, but no difference in the incidence of wound infection or grafting was found.^22^

Cubo gels were reported to be effective in treatment of deep second degree burns which may result into better patient compliance and excellent healing results with least side effects in comparison with the commercially available products.^23^ As thermal injury disrupts the protective barrier function of skin, dressing is needed to protect against environmental flora and evaporative heat loss. But, it can clean the wound and remove the debries of the separated eschar and devitalized tissue and has an antibacterial activity. 

A wide variety of substances have been reported to be useful in the treatment of burn wounds.^24^ Among antimicrobial agents, topical ointment of silver sulfadiazine is the most commonly deployed for partial and full-thickness burns.^25^ In another study, silversulfadiazin and aloevera were compared and reported a higher healing speed in aloevera group.^26^ In the present study, nettle extract was compared with vaseline ointment and silver sulfadiazine as the standard treatment for burn wound in rats. 

Stinging nettle has been used to treat painful muscles and joints, eczema, arthritis, gout, anemia, benign prostatic hyperplasia, urinary tract infections, hay fever (allergic rhinitis) and thumb pain. It has also been used for treating joint pain, sprains and strains, tendonitis and insect bites.^12^^-^^15^ IDS 23, a stinging nettle leaf extract, may inhibit inflammatory cascade in autoimmune diseases.^27^ Many traditional plants have been used for repair of burn wounds in animal models; however, no specific surveys have been done about effect of nettle extract on healing of burn wounds. 

The probable mechanisms include providing necessary materials for healing, increasing blood flow to burn area, decreasing the inflammatory response and decreasing the rate of infection. In the histological evaluation, maximal rate of angiogenesis and fibrosis were seen in nettle group with a better wound healing. Our findings showed the maximal rate of healing in the nettle group. So it may be a suitable substitute for silver sulfadiazine and vaseline when available.

## Note

Please cite this paper as:

Akbari H, Fatemi MJ, Iranpour M, Khodarahmi A, Baghaee M, Pedram MS, Saleh S, Araghi S. The Healing Effect of Nettle Extract on Second Degree Burn Wounds. World J Plast Surg 2015;4(1):23-28.
